# The Effect of Tong-Xie-Yao-Fang on Intestinal Mucosal Mast Cells in Postinfectious Irritable Bowel Syndrome Rats

**DOI:** 10.1155/2017/9086034

**Published:** 2017-02-26

**Authors:** Xiangxue Ma, Xiaoge Wang, Nan Kang, Ting Chen, Haijie Ji, Lin Lv, Xiaolan Yin, Yaxin Tian, Rui Zheng, Yuanzhi Duan, Fengyun Wang, Xudong Tang

**Affiliations:** ^1^Graduate School, Beijing University of Chinese Medicine, No. 11, Bei San Huan Dong Lu, Chaoyang District, Beijing 100029, China; ^2^Gastroenterology Department, Xiyuan Hospital, China Academy of Chinese Medical Sciences, 1 Xi Yuan Yard Road, Haidian District, Beijing 100091, China; ^3^The First Affiliated Hospital of Henan University of TCM, Zhengzhou, Henan 450000, China; ^4^Department of Pharmaceutical Preparation, Xiyuan Hospital, China Academy of Chinese Medical Sciences, 1 Xi Yuan Yard Road, Beijing 100091, China

## Abstract

*Objective*. To investigate the effects of Tong-Xie-Yao-Fang (TXYF) on intestinal mucosal mast cells in rats with postinfectious irritable bowel syndrome (PI-IBS).* Design*. PI-IBS rat models were established using a multistimulation paradigm. Then, rats were treated with TXYF intragastrically at doses of 2.5, 5.0, and 10.0 g·kg^−1^·d^−1^ for 14 days, respectively. Intestinal sensitivity was assessed based on abdominal withdrawal reflex (AWR) scores and fecal water content (FWC). Mast cell counts and the immunofluorescence of tryptase and c-Fos in intestinal mucosa were measured; and serum IL-1*β*, TNF-*α*, and histamine levels were determined.* Results*. AWR reactivity and FWC which were significantly increased could be observed in PI-IBS rats. Remarkably increased mast cell activation ratio in intestinal mucosa, together with increased serum TNF-*α* and histamine levels, could also be seen in PI-IBS rats; furthermore, PI-IBS-induced changes in mast cell activation and level of serum TNF-*α* and histamine could be reversed by TXYF treatment. Meanwhile, tryptase and c-Fos expression were also downregulated.* Conclusion*. TXYF improves PI-IBS symptoms by alleviating behavioral hyperalgesia and antidiarrhea, the underlying mechanism of which involves the inhibitory effects of TXYF on activating mucosal mast cells, downregulating tryptase and c-Fos expression, and reducing serum TNF-*α* and histamine levels.

## 1. Introduction

Irritable bowel syndrome (IBS) is a common gastrointestinal disorder that manifests as abdominal discomfort and altered bowel habits, but IBS-related abnormalities are not obvious during routine diagnostic tests [1]. IBS-related symptoms in some patients may be secondary to acute gastroenteritis, and such phenomenon is known as postinfectious IBS (PI-IBS), which exhibits characteristics that are similar to those of diarrhea-predominant IBS (IBS-D), accounting for 4% to 31% of IBS patients [2–5]. Thus, PI-IBS has attracted growing attention due to its clear onset and well-defined pathophysiological changes, which may help to explain the occurrence of IBS [6].

Mast cells are immune cells that are widely distributed in the gastrointestinal tract, the cytoplasm of which is rich in endocrine granules that will synthesize and release various bioactive media and factors in response to stimuli, including histamine, 5-hydroxytryptamine (5-HT), tryptase, prostaglandins, and cytokines. The extensive actions of these active media will increase responses of the enteric nervous system and irritate the sensory afferent nerve pathways thus inducing visceral hyperalgesia and intestinal kinetic imbalance. As is reported previously [7, 8], the number of intestinal mucosal mast cells and tryptase levels in IBS patients are higher than those in healthy subjects, among which, the proliferation and activation of colonic mast cells are positively correlated with the degrees of abdominal pain and abdominal distension in IBS patients [9]. Intestinal mucosal mast cells are close to the route of intestinal nerve fibers in position; furthermore, membranes of a small amount of mast cells are even connected to those of nerve fiber cells, thus rendering a pivotal role of mast cells in the occurrence of PI-IBS [10].

Tong-Xie-Yao-Fang (TXYF), a prescription in traditional Chinese medicine (TCM) that has been extensively applied in relieving IBS-associated symptoms since the Yuan Dynasty [11], is comprised of* Atractylodes rhizome*,* white peony root, dried old orange peel,* and* ledebouriella root*. It has been demonstrated in preliminary experiments that TXYF has significant analgesic effects on IBS rats by regulating 5-HT in the periphery and corticotropin-releasing factor (CRF) in the center [12]. TXYF has been shown in a preliminary clinical study to demonstrate an antipain and antidiarrhea effect on IBS-D patients [13]; besides, it can also regulate cytokine levels in the colonic mucosa, which may account for a potential molecular mechanism of its effect on IBS-D [14]. However, the precise mechanism underlying the action of TXYF remains to be fully illuminated yet.

Thus, further investigation may provide insights into the systemic pharmacological repair mechanisms of TXYF. The present study aimed to evaluate the effects of TXYF on mast cell activation in PI-IBS rat models.

## 2. Materials and Methods

### 2.1. Animals

Adult pregnant Sprague-Dawley rats with the weight of 220 to 230 g, which were provided by Beijing Weitonglihua Experimental Animal Technology Co., Ltd. (Beijing, China), were housed in the animal room of laboratory and maintained under the following conditions: the temperature of 23 ± 2°C, the humidity of 65% ± 5%, and a 12 h/12 h light/dark cycle (lighting from 7:30 to 19:30). The animals were provided with food and water ad libitum. Only male newborn rats were used. All experimental protocols described here were approved by the Ethics Review Committee at Animal Experimentation of Xiyuan Hospital, China Academy of Chinese Medical Sciences.

### 2.2. Experimental Medicine

The ingredients of TXYF were shown as follows:* Rhizoma Atractylodis macrocephalae* (18 g),* Radix Paeoniae alba *(12 g),* Pericarpium citri reticulatae *(9 g), and* Radix saposhnikoviae* (6 g), which were the daily clinical doses for humans. TXYF was prepared by the Department of Pharmaceutical Preparation of Xiyuan Hospital in accordance with good manufacturing practices and was maintained at room temperature. All herbs were purchased from qualified suppliers in China.

Both disodium cromoglycate (DSCG) and 2,4,6-trinitrobenzenesulfonic acid solution (TNBS) were bought from Sigma-Aldrich (St. Louis, MO, USA). Interleukin- (IL-) 1*β* and tumor necrosis factor- (TNF-) *α* enzyme-linked immunosorbent assay (ELISA) kits were obtained from SenXiong Bioengineering Institute (Shanghai, China). Histamine ELISA kit was purchased from GenWay Biotech Inc. (Ebioscience, San Diego, CA, USA).

### 2.3. Experimental Design

This experiment aimed to test whether TXYF could inhibit mast cell activation in PI-IBS rat models. Rats were randomly divided into 6 groups with 8 rats in each group, namely, normal group in which the normal rats were treated with saline, as well as model group, TXYF-L, TXYF-M, TXYF-H, and DSCG groups, in which PI-IBS rats were treated with saline, low-dose TXYF, medium-dose TXYF, high-dose TXYF, and DSCG, respectively. Rats in TXYF medication groups were administered with TXYF at doses of 2.5, 5.0, and 10 g/kg intragastrically (ig) once daily for 14 consecutive days. Normal and model groups were administered with saline. The doses of TXYF used here were calculated according to the clinical doses of raw materials. Abdominal withdrawal reflex (AWR) and fecal water content (FWC) in rats were measured after 2 weeks of treatment; subsequently, all rats were sacrificed to collect colons. Each segment of proximal colon (4 cm in length; 1-2 cm away from caecum) was harvested and divided into 2 parts, with the proximal part being used for mast cell observation and the transverse one for MCT and c-Fos immunofluorescence assay. Serum was collected for detecting IL-1*β*, TNF-*α*, and histamine levels.

### 2.4. Neonatal Maternal Separation (NMS)

Neonatal maternal separation (NMS) was performed according to a reported procedure [15, 16]. Briefly, litters were removed from their maternity cages to adjacent cages at 9:00–12:00 am from day 2 postnatally (abbreviated as PN2, the same below) to PN14, and those in normal group were housed together with their mothers. Pups with the same sex or in the same group were housed together after they were weaned (with 4 pups in a cage). Male adult pups from NMS group were randomly divided into 5 groups (*n* = 8) on PN30, which were PI-IBS model group, PI-IBS + TXYF-H group (10 g/kg), PI-IBS + TXYF-M group (5 g/kg), PI-IBS + TXYF-L group (2.5 g/kg), and PI-IBS + DSCG group (0.1 g/kg).

### 2.5. Induction of Colitis Using TNBS

Colitis was induced in rats after pentobarbital anesthesia based on a previously published method, which was intrarectal administration of 0.8 mL TNBS solution (5 mg per rat) in 50% ethanol on PN42 [17]. The control rats were given 0.8 mL of 50% ethanol vehicle. All solutions were delivered via a soft catheter introduced 8 cm beyond the anus.

### 2.6. Chronic Unpredictable Mild Stress (CUMS)

After recovery from TNBS modeling for 2 weeks, CUMS modeling was performed as follows [18, 19]: (1) water-fasting for 24 h, (2) fasting for 24 h, (3) reverse day/night cycle (dark treatment from 7:00 to 19:00, lighting from 19:00 to 7:00 on next day), (4) cold stress (rats were put into a transparent barrel containing ice water at 4°C at the depth of 15 cm for 5 min), (5) heat stress (rats were put into a thermostat at 45°C for 5 min), (6) pain induction (rats were put into an observation cage, the tails of which were clipped at 1 cm from the distal tip, and the strength should be as appropriate as to make the rat scream), and (7) horizontal oscillation (rats were placed into a horizontal oscillator at high-speed (110/min) for 15 min). Each type of stress was performed daily for 21 consecutive days.

### 2.7. AWR

Colorectal distension (CRD) was administered as previously described one day after CUMS and one day after TXYF medication, and AWR scores of all rats were quantified [20]. Specifically, a latex double-lumen catheter attached to a balloon dilator (2 mm in diameter) was used. The balloon was vaseline-coated and inserted into the descending colon with the distal tip locating at the site 8 cm away from the anus, and CRD was maintained by water injection. The rats were placed in small lucite cubicles and allowed to wake up and adapt for 15 min. CRD was repeated for 3 times to achieve an accurate measurement. AWR responses were measured by blind observers as according to the following standard: 0 points: no behavioral response to CRD; 1 point: simple head movement followed by immobility; 2 points: contraction of abdominal muscles; 3 points: lifting of the abdomen; and 4 points: arching of body and lifting of pelvic structures. CRD was calculated as the amount of injected water when the AWR score was 3.

### 2.8. Open-Field Test

Rats were placed individually in the middle of an open-field apparatus (height: 40.0 cm; length: 100.0 cm; width: 100.0 cm). Specifically, 25 squares (20.0 cm × 20.0 cm) were delineated on the floor, and the number of times when the rat crossed between squares was measured by two observers independently who were blind to the experimental groups over a 10 min period [21, 22]. Crossing, a measure of locomotion, was quantified when the rat moved all four legs from one quadrant to another. The open-field apparatus was carefully cleaned after each trial.

### 2.9. Measurement of Serum IL-1*β*, TNF-*α*, and Histamine Levels

Serum IL-1*β*, TNF-*α*, and histamine levels were measured using commercial ELISA kits according to the manufacturer's instructions.

### 2.10. Mast Cell Counts

Proximal colonic tissues were prepared as paraffin slices (5–10 um), which were then dewaxed and hydrated; subsequently, they were soaked in 70% alcohol for 1-2 min, followed by staining with dripping toluidine blue for 5–10 min and washing with tap water; later they were rapidly dehydrated with acetone for 20–30 s, treated with dimethylbenzene for vitrification and sealed with neutral gel. Three fields of view (FOV) were randomly selected from each slice. Next, 6 rats from each group were selected, immune-positive cells were determined on Image Pro Plus 6.0, and images were obtained using a 400x microscope. Five FOVs were randomly selected from each slice to determine the average count of positive cells.

### 2.11. Immunofluorescence of MCT and c-Fos

Whole-mount preparations of slices were incubated in 10% normal donkey serum in PBS for 30 minutes at room temperature to suppress nonspecific binding of immunoglobulin. The tissues were then placed in a humidified chamber for double-immunofluorescence staining, which was processed as follows: incubating the tissue in the mixture of primary antibodies (anti-MCT antibody and anti-c-Fos antibody) from different species for 24 hours at room temperature for double labeling. After being incubated with the primary antibodies, the tissues were washed with PBS for 5 min for 3 times, transferred to a humidified chamber, and incubated in the mixture of appropriate secondary antibodies at room temperature for 1 hour. The double-labeling sections were incubated with fluorescent goat anti-rabbit fluorescein isothiocyanate (FITC) and goat anti-mouse TRITC secondary antibody. Tetramethyl rhodamine iso-thiocyanate- (TRITC-) and fluorescein isothiocyanate- (FITC-) marked antigens (TRITC-marked red cells were MCT-positive cells, and FITC-marked green cells were c-Fos-positive cells) in slice tissues were observed under fluorescence microscope at the wavelength of 546 and 490 nm, respectively, and images at 400x fluorescence microscope were obtained.

### 2.12. Data Quantification and Statistical Analysis

The data were expressed as *x* ± *s*. Data conforming to normal distribution and variance homogeneity were tested via one-factor analysis of variance (ANOVA) and compared in pairs by least significant difference (LSD). Data not according with normal distribution or variance homogeneity were assessed using the multiple independent-sample Kruskal-Wallis *H* test (a nonparametric test) and compared in pairs using the Kruskal-Wallis single-factor ANOVA. The same group before and after intervention was assessed via paired *T*-test. Significance level was set at *P* < 0.05.

## 3. Results

### 3.1. Weight

**Figure 1:**
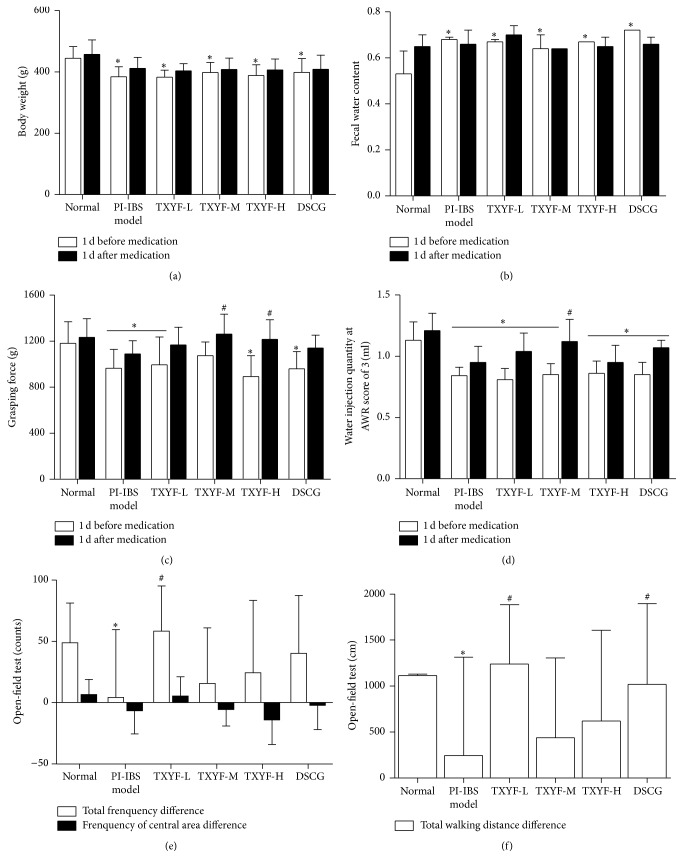
Effects of TXYF on body weight, fecal water content, grasping force, AWR, and locomotor activity in PI-IBS rats. (a) Rats' body weight, (b) fecal water content, (c) grasping force, (d) water injection quantity at an AWR score of 3, and (e, f) locomotor activity in control and TXYF treated groups. Mean ± SD. *n* = 8. ^*∗*^*P* < 0.05 versus normal. ^#^*P* < 0.05 versus model.

The weight in all medication groups and model groups before medication was significantly lower than that in normal group (*P* < 0.05), while that in each group after medication increased relative to that before medication, but the difference between all medication groups and model group was of no statistical significance (*P* > 0.05) (Figure 1(a)).

### 3.2. FWC

Fecal water content of rats in all medication groups and model group was notably higher than that in normal group before medication. In terms of intragroup comparison before and after medication, fecal water content in each medication group was lower after medication than before, except for the TXYF-L group, and changes in sodium cromoglycate group were the most obvious (*P* < 0.05). But the difference in fecal water content between all medication groups and model group was of no statistical significance after medication (Figure 1(b)).

### 3.3. Grasping Force

Grasping force of rats in all medication groups was lower than that in normal group before medication (*P* < 0.05); that in model group was markedly lower than that in normal group after medication (*P* < 0.05); compared with model group, that in TXYF-H group and TXYF-M was distinctly improved (all *P* < 0.05), and difference in grasping force between TXYF-L group as well as DSCG group and model group was of no statistical significance (*P* > 0.05). It suggested that high- and moderate-dose TXYF could improve the grasping force in rats (Figure 1(c)).

### 3.4. AWR

AWR of rats in each group was outstandingly lower than that in normal group before medication (*P* < 0.05); compared with model group, that in TXYF-M group and DSCG group was remarkably increased (both *P* < 0.05) after medication. When comparing the difference before and after medication, that in TXYF-M and TXYF-L groups, together with DSCG group, was notably increased after mediation than before (*P* < 0.05), suggesting that all medication groups had increased AWR in PI-IBS model rats, with effects of TXYF-M being the most outstanding (Figure 1(d)).

### 3.5. Open-Field Test

Results of open-field test revealed that model group was markedly lower than normal group (*P* < 0.05), while TXYF-L group was remarkably higher than model group (*P* < 0.05) in terms of total frequency difference, indicating that low-dose TXYF could increase the total frequency difference. From the point of view of total crossing distance, that in model group was outstandingly lower than that in normal group (*P* < 0.05), and that in TXYF-L group and DSCG group was apparently higher than that in model group (both *P* < 0.05), demonstrating that low-dose TXYF and DSCG could enhance total crossing distance. There was no significant difference with regard to number of crossings in the middle area. Low-dose TXYF and DSCG could increase total number of crossings and total crossing distance in PI-IBS model rats, and the former had superior effects to the latter (Figures 1(e) and 1(f)).

### 3.6. ELISA of IL-1*β*, TNF-*α*, and Histamine

**Figure 2:**
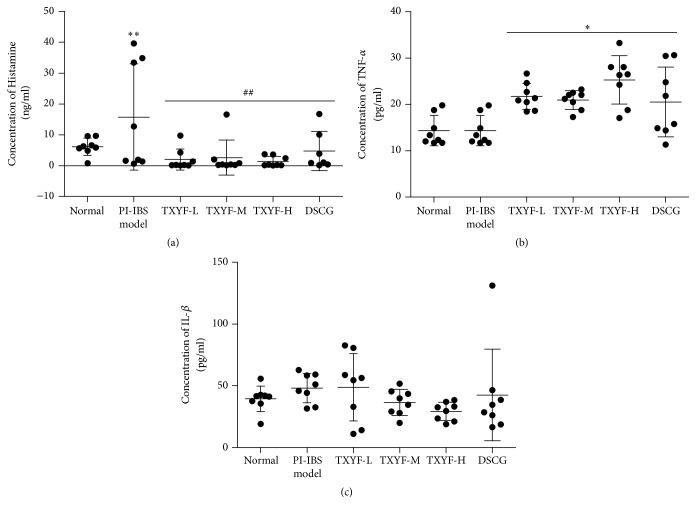
Histamine, TNF-*α*, and IL-1*β* expression in PI-IBS rats serum. Data are presented as the mean ± SD. *n* = 8. ^*∗*^*P* < 0.05; ^*∗∗*^*P* < 0.01 versus normal; ^##^*P* < 0.01 versus model.

*(1) Histamine Levels.* Serum histamine levels were significantly higher in the model group compared with the normal group (*P* < 0.05) and were significantly smaller in the TXYF-H, TXYF-M, TXYF-L, and DSCG groups compared with the model group (all *P* < 0.05). This finding indicated that three-factor modeling significantly improved the serum histamine contents in rats, and the TXYF-H, TXYF-M, TXYF-L, and DSCG groups significantly reduced serum histamine contents in the model group. There was no significant difference among the medicated groups (Figure 2(a)).

*(2) TNF-α Levels.* Serum TNF-*α* levels were significantly increased in the model group and all medicated groups compared with the normal group (all *P* < 0.05). Three-factor modeling (NMS + TNBS + CUMS) enhanced the action of rat serum TNF-*α*, which was not significantly affected by any of the medications (Figure 2(b)).

*(3) IL-β Levels.* No significant difference was found between any two groups (*P* > 0.05), indicating that three-factor modeling, TXYF, or DSCG does not significantly affect serum IL-*β* levels in rats (Figure 2(c)).

### 3.7. Mast Cell Staining Count

**Figure 3:**
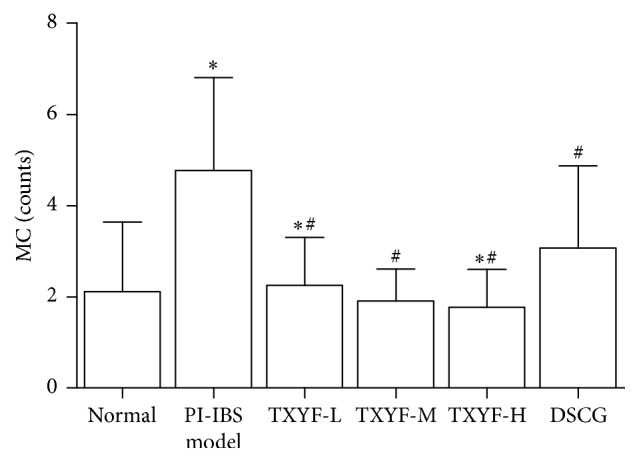
Mast cells counts at each group rats' proximal colon. mean ± SD. *n* = 8. ^*∗*^*P* < 0.05 versus normal. ^#^*P* < 0.05 versus model.

**Figure 4:**
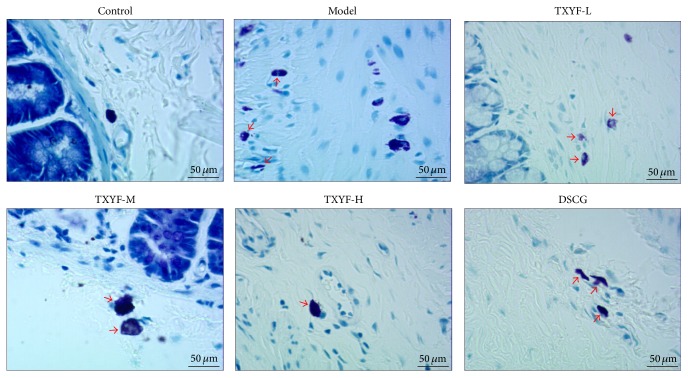
Mast cells were detected with toluidine blue stain (the red arrow indicated the mast cells).

Results of toluidine blue staining indicated that dispersedly distributed mast cells (MCs) could be seen in lamina propria and submucosa of proximal colonic mucosa under light microscope; the MCs were oval or fusiform, with the cytoplasm being stained purple and nucleus blue; those without degranulation were intact, with uniform cytoplasm that was stained purple, while those with degranulation had irregular morphology, with lighter cytoplasm color, and purple granular materials could be seen around cells. Average MC count under each high power field (×400) is as follows: MC count in proximal colon in model group was remarkably increased relative to normal group (*P* < 0.01); compared with model group, that in TXYF-M and TXYF-L groups and DSCG group was notably decreased (*P* < 0.01), while that in TXYF-H and TXYF-M groups was distinctly lower than that in DSCG group (*P* < 0.05). It is suggested that three-factor modeling could remarkably enhance MC count in proximal colonic mucosa of rats (*P* < 0.01) and that high and moderate TXYF, as well as sodium cromoglycate, could significantly reduce colonic mucosa MC count in PI-IBS rats (*P* < 0.01), with the effects of high- and moderate-dose TXYF being superior to those of DSCG (Figures 3 and 4).

### 3.8. MCT and c-Fos Detected via Immunofluorescence

**Figure 5:**
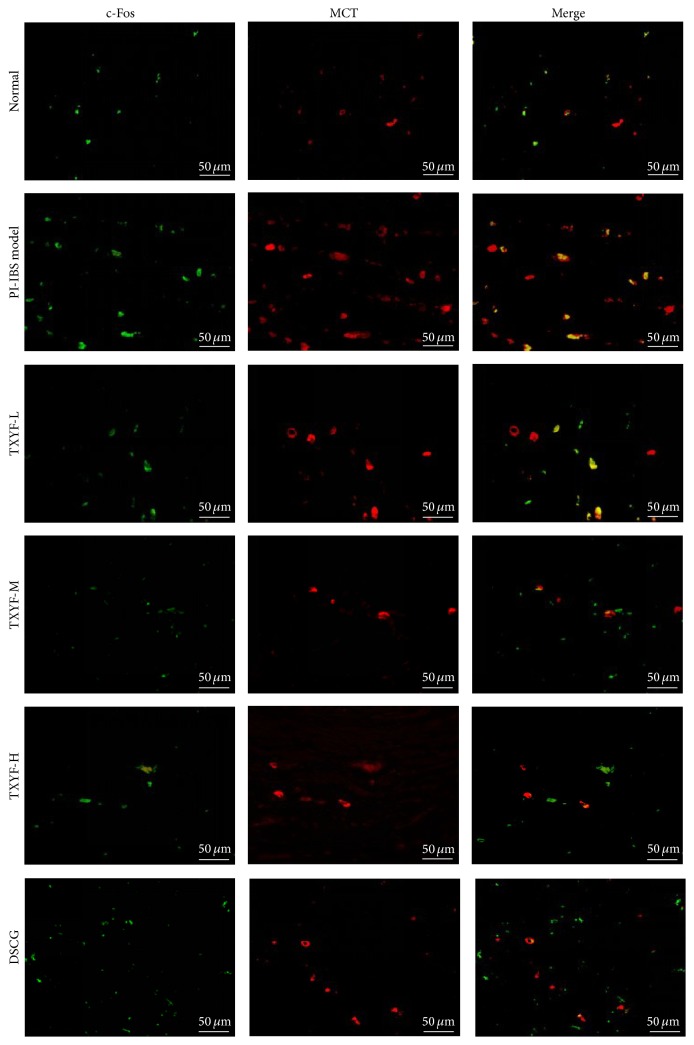
Expression of MCT and c-Fos in the lamina propria and submucosa of the proximal colon by double-label immunofluorescence assays.

**Figure 6:**
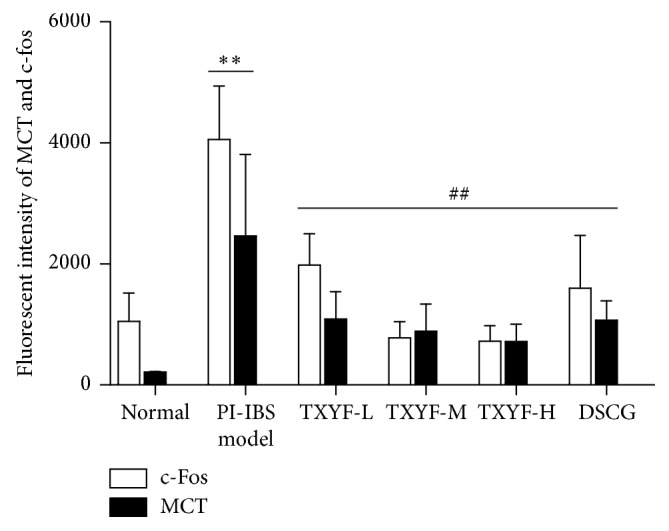
The fluorescent intensity of MCT and c-Fos in control and treated groups. Mean ± SD. *n* = 3. ^*∗∗*^*P* < 0.01 versus normal; ^##^*P* < 0.01 versus model.

Dispersedly distributed MCT positive cells (red fluorescence) could be seen in lamina propria and submucosa of proximal colonic mucosa under fluorescence microscope; MCT was mainly expressed in the cytoplasm, and MCT positive cells were mostly ring-shaped under fluorescence microscope since nuclei in the center of cells could not be stained, while c-Fos protein was mainly expressed in nuclei of MCT positive cells or in nuclei of surrounding neurons (green fluorescence). Under each high power field (×400), fluorescence optical densities of MCT and c-Fos in model group were remarkably higher than those in normal group (*P* < 0.05); fluorescence optical density of c-Fos in TXYF-L, TXYF-M, and TXYF-H groups, as well as DSCG group, was markedly lower than that in model group (all *P* < 0.01); that of MCT in TXYF-L, TXYF-M, and TXYF-H groups, as well as DSCG group, was outstandingly lower than that in model group (all *P* < 0.01), but the difference in all medication groups was of no statistical significance. It is suggested that low-, moderate-, and high-dose TXYF, as well as DSCG, could inhibit expression of MCT and c-Fos (Figures 5 and 6).

## 4. Discussion

It is indicated in this research that TXYF contributes to relieving the visceral hypersensitivity in PI-IBS rat models by inhibiting the activation of mast cells and regulating the expression of MCT and c-Fos, as well as the levels of TNF-*α* and histamine.

Though the detailed pathological mechanism of IBS remains unclear currently, both stress and psychological factors are regarded as essential factors affecting IBS [23]. In addition, low-grade inflammation has been increasingly recognized to play a significant role in the development of IBS secondary to acute gastroenteritis [24, 25]. Meanwhile, complex interactions among gut, immune system, and nervous system will impact the development and progression of IBS, as is suggested in previous research; therefore, an appropriate animal model is necessary to accurately describe the pathophysiological process of IBS. It has been suggested in previous studies that PI-IBS rat model can be developed with stimulation of 3-factor modeling (namely, NMS, TNBS, and CUMS) in accordance with the related symptoms, including visceral hypersensitivity, high fecal water content, anxiety, depression, and mild colitis [16]. Therefore, 3-factor modeling rats can adequately reflect the typical characteristics of PI-IBS.

As is reported previously, compared with control group and noninfected IBS tissues, increased mast cell counts can be seen in duodenum, jejunum, distal ileum, colon, and rectum mucosa of PI-IBS patients; the increasing degree of which is positively correlated with the severity of IBS symptoms [26]. Mast cells, which may play a vital role in the pathophysiological process of IBS, are probably an intermediate medium affecting the interrelation and interaction between the intestinal tract and nervous system. Mast cell-derived active substances, such as histamine, 5-HT, tryptase, prostaglandins, cytokines, and leukotriene, which are most likely to act on the enteric nervous system, can potentially activate visceral afferent nerves [27], resulting in gastrointestinal discomfort and hypersensitivity [28]. Among all substances released by mast cells, tryptase, which is stored in small particles of mast cell cytoplasm, may play a specific role in the transmission of neuronal information. As a major bioactive molecule and a specific mark of activating degranulation [29], tryptase can act in a way that is similar to the activated mast cells [30] that can secrete inflammatory media TNF-*α* and IL-1*β*. Systemic evaluation of clinical literature indicates that, compared with normal participants, IBS patients have distinctly increased serum TNF-*α* and IL-1*β* levels; particularly, IL-1*β* level is notably increased in PI-IBS patients [31]. In addition to remarkably increased IL-1*β* level in PI-IBS patients, TNF-*α* level is also significantly elevated in IBS patients [32]. TNF-*α* and IL-6 are regarded as the most important inflammatory cytokines in IBS patients. Outstandingly elevated levels of IL-8, IL-10, IL-1*β*, and TGF-1*β* can be observed in IBS patients, among which, distinctly elevated IL-1*β* expression can also be seen in PI-IBS patients particularly [33].

c-Fos, a nuclear phosphorylated protein, is mainly activated and transcriptionally translated by c-Fos protooncogene in nucleus after cell irritation. c-Fos protooncogenes are usually poorly expressed; however, they can be briefly and quickly expressed upon the onset of external stimuli, so as to synthesize c-Fos protein, thus playing important roles in cell proliferation, differentiation, and signal conduction. As is reported recently, expression of c-Fos protein in the central nervous system and gastrointestinal intermuscular neural cells is crucial in the transfer of visceral pain information [34, 35]. c-Fos expression in mast cells also plays a key role in the proliferation and signal conduction of mast cells [36].

IBS-D belongs to the category of “diarrhea,” “abdominal pain,” and “depression disease” in accordance with TCM classification; one major pathogenesis of which is liver-spleen disharmony. TXYF, a representative prescription for the treatment of abdominal pain and diarrhea, is consistent with the rules of “regulating liver functions and rectifying the spleen,” and thus it is widely applied in the clinical treatment of IBS-D. PI-IBS rat models in this study are associated with symptoms such as visceral hypersensitivity, high FWC, anxiety, depression, and slight colitis, which are coincided with liver-qi stagnation and spleen deficiency in TCM.

The experimental results show that proximal colonic mast cell counts are significantly greater in PI-IBS rats compared with normal rats, which is consistent with results in the existing research. Both TXYF and DSCG can reduce mast cell counts, and, specifically, both high-dose and medium-dose TXYF are more effective than DSCG. ODs of c-Fos and MCT are both remarkably higher in model group relative to normal group, but they are markedly lower in all medication groups compared with model group. The histamine level detected by ELISA is significantly higher in model group compared with normal group, but that is significantly smaller in all medication groups when comparing to model group, indicating that TXYF can significantly reduce serum histamine level in PI-IBS rats. DSCG is a blocking agent against mast cell degranulation, which inhibits Ca^2+^ from entering mast cells and contributes to temporally stabilizing mast cells without degranulation [37, 38], thus exerting an inhibitory effect on inflammatory and immune responses. DSCG, which is originally used to treat bronchial asthma, has been shown in recent studies to significantly block intestinal mast cell degranulation. Thus, DSCG is selected as a positive control in this paper. We find that DSCG has remarkable inhibitory effects on the proliferation of mast cells in PI-IBS rats; furthermore, MCT, c-Fos, and histamine levels are related to the activation of mast cells. In comparison, TXYF outperforms DSCG in reducing the proliferation of mast cells, but there is no significant difference in the inhibition of mast cell degranulation between the two. Serum TNF-*α* level in PI-IBS rats is significantly elevated, but TXYF shows no significant effect on it. No significant difference in IL-*β* level can be seen among all groups, indicating that none of model, TXYF, or DSCG can significantly affect serum IL-*β* level in rats. In conclusion, the PI-IBS rat model developed in this study is characterized by visceral hypersensitivity, the intestinal dynamic changes of which are consistent with the manifestations of PI-IBS, thus demonstrating that it is an ideal model. The effects of different doses of TXYF on the manifestations and serological indices in model rats are predominantly consistent with those in other studies. Moreover, TXYF can significantly inhibit the proliferation and degranulation of colonic mucosal mast cells in model rats; besides, it has higher overall therapeutic effects than those observed in DSCG.

## 5. Conclusions

TXYF improves PI-IBS symptoms by relieving behavioral hyperalgesia and antidiarrhea, the underlying mechanism of which involves the inhibitory effects of TXYF on activating mucosal mast cells, downregulating tryptase and c-fos expression, as well as reducing serum TNF-*α* and histamine levels.
